# Conferred with a new life: A case report of management of a severe midface trauma and subsequent oral rehabilitation

**DOI:** 10.1002/ccr3.4620

**Published:** 2021-08-25

**Authors:** Alireza Pournabi, Hamidreza Moslemi, Shervin Shafiei, Ramtin Dastgir, Kamyar Abbasi, Mostafa Alam

**Affiliations:** ^1^ Oral and Maxillofacial Surgeon Qaemshahr Iran; ^2^ Department of Oral and Maxillofacial Surgery School of Dentistry Shahid Beheshti University of Medical Sciences Tehran Iran; ^3^ Faculty of Dentistry, Tehran Medical Sciences Islamic Azad University Tehran Iran; ^4^ Department of Prosthodontics School of Dentistry Shahid Beheshti University of Medical Sciences Tehran Iran; ^5^ Department of Oral and Maxillofacial Surgery School of Dentistry Shahid Beheshti University of Medical Sciences Tehran Iran

**Keywords:** alveolar ridge augmentation, dental implants, maxillofacial injuries, quality of life

## Abstract

Maxillofacial traumas have been associated with 14%‐17% of all facial injuries. The most common etiology of mid‐facial traumas is motor vehicle accidents followed by interpersonal assaults. The devastating nature of maxillofacial defects makes reconstruction of the midface challenging, due to multiple required surgeries and extensive rehabilitation phase. The midface has been defined as the area between the zygomaticofrontal sutures and the maxillary occlusal plane. Midface traumas are significantly more challenging to manage compared to isolated facial since there is limited intact and unharmed framework to guide with anatomic reductions. Therefore, the appropriate surgical approach to a maxillofacial trauma must follow a systematic scheme. Besides, one of the main consequences of maxillofacial traumas is destruction of the teeth and teeth bearing alveolar bone. Oral rehabilitation utilizing dental implants of these patients must be considered to provide the higher quality of life. Here, we report the management and further oral rehabilitation of a case suffering severe midface trauma following a motor vehicle accident where the patient was hit by a lorry.

## Graphical abstract

In this study, we reported the management and further oral rehabilitation of a case suffering severe midface trauma. This study emphasizes on the crucial role of a well‐managed surgery and its subsequent improvement in patient’s quality of life.

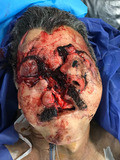

## 1 INTRODUCTION

A person’s countenance depends directly on the skeletal architecture and the overlying soft tissues of the midface ^1^. The midface has been defined as the area between the zygomaticofrontal sutures and the maxillary occlusal plane. These planes converge posteriorly meeting near the foramen magnum. This region encompasses the entire maxilla, zygomatic bones, and nasal orbital ethmoid (NOE) complex along with the nasal substructures. This region holds a high density of vascular, musculoskeletal, and nervous system structures whose injury will often result in substantial morbidity and mortality in cases of severe midface traumas. A comprehensive knowledge of head and neck skeletal and soft tissue anatomy is imperative in understanding how to manage the patients presenting with these patterns of traumas ^2^. These traumas are significantly more challenging to manage compared to isolated facial or dentoalveolar fractures since there is limited intact and unharmed framework to guide with anatomic reductions. Furthermore, oftentimes, due to the high impact of force that caused these traumas in the first place, these patients present with other significant concomitant traumas that must be managed concurrently ^3^. The appropriate surgical approach to a maxillofacial trauma must follow a systematic scheme, necessitating systemic evaluations such as the hemodynamic evaluation, wound extensions, presence or absence of foreign bodies, neural or vascular or glandular ducts involvements, and other requisite evaluations which must be carefully undertaken with preoperative examinations ^4^. Inability to directly visualize and reduce all the components of a mid‐facial injury along with inadequate stability of the fractured skeletal compartments leads to postoperative deformity. Each case with this type of fracture is unique and requires skill and expertise of the surgeon to restore the pre‐traumatic facial anatomy, esthetics, and functions ^5^. In this article, we report the management and further oral rehabilitation of a case suffering severe midface trauma following a motor vehicle accident were the patient was hit by a commercial lorry.

## 2 CASE PRESENTATION

**FIGURE 1:**
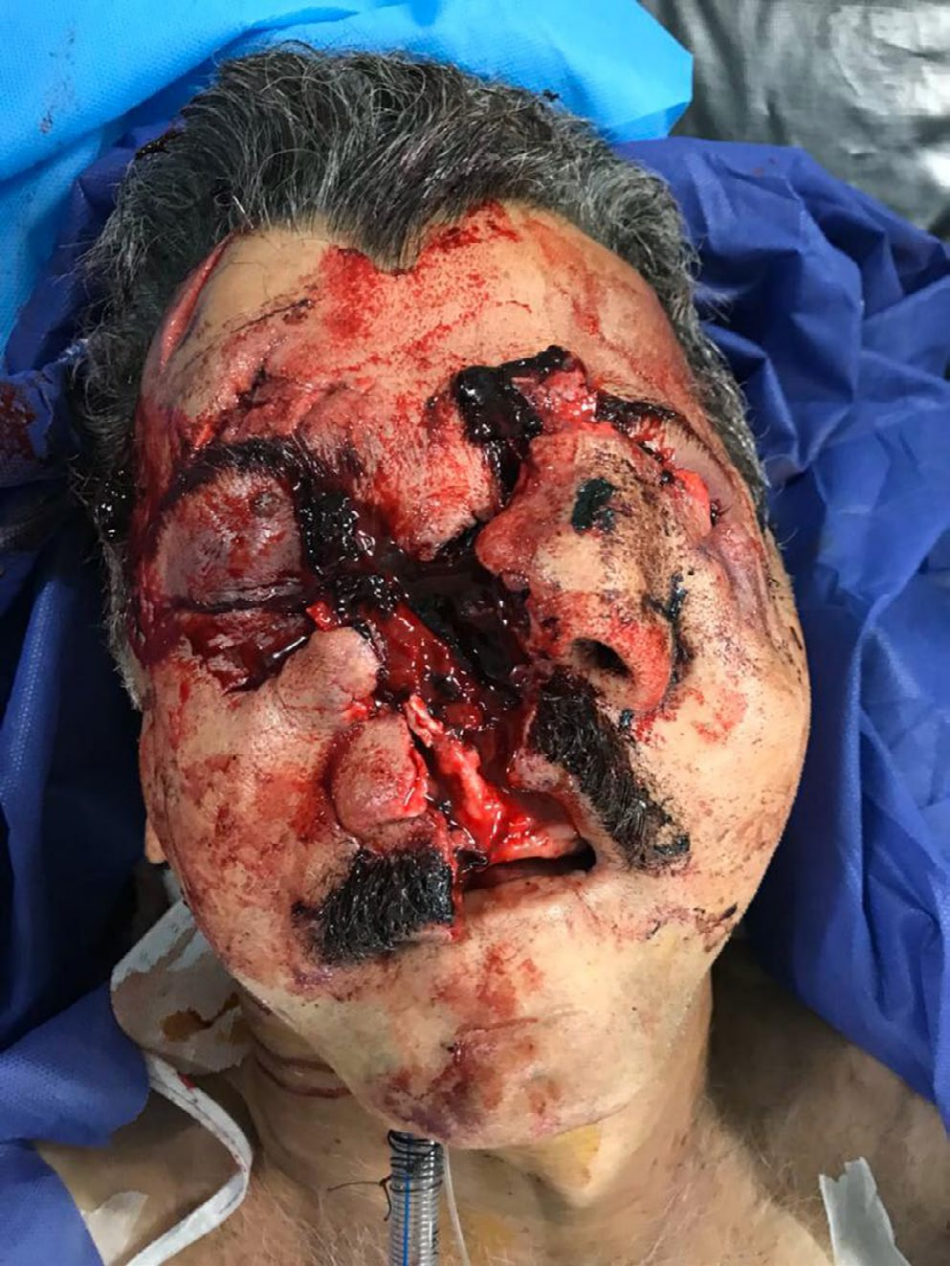
Patient presenting to the emergency department; submental intubation acquired

A 53‐year‐old man was presented to the emergency department of Shahid Beheshti hospital in Babol, Iran, following a motor vehicle accident involving the patient getting hit by a high‐velocity lorry as he was crossing the street. Upon presenting to the emergency department, neurological and surgical evaluations were done and intracranial, cervical spine, thoracic, abdominal, and other severe extremity injuries were ruled out. He had sustained soft tissue lacerations extending from supraorbital regions to upper lip with almost complete destruction of nasal structures and hemorrhage (Figure 1). Furthermore, intraoral examinations revealed intact maxillary edentulous and mandibular partially edentulous ridge. A computerized tomography (CT) scan with three‐dimensional reconstruction was prompted and it further depicted the extent of the injury. No concomitant injuries in upper and lower extremities were observed. Preoperative laboratory tests revealed normal findings.

**FIGURE 2:**
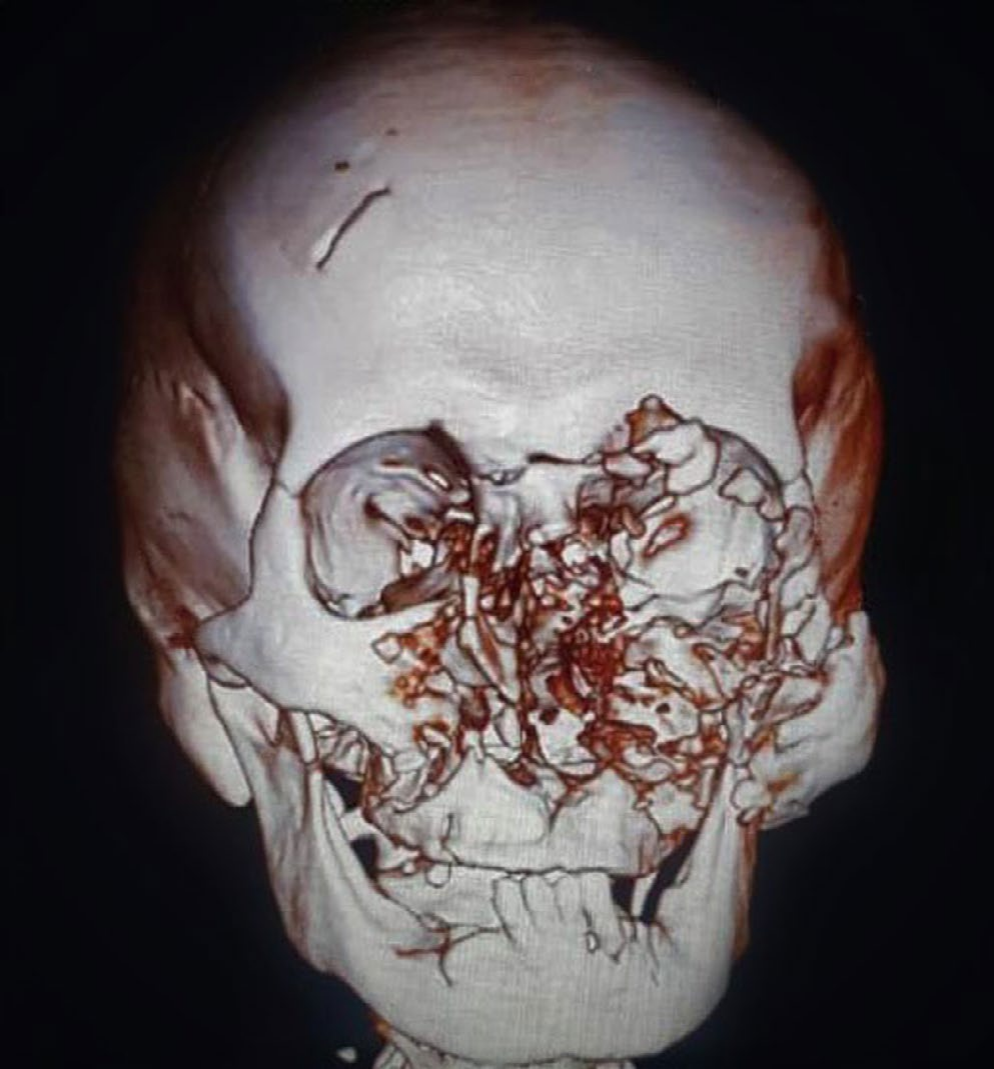
Preoperative three‐dimensional CT scan depicting the extent of fractures

Regarding CT scan analysis, left orbital floor was completely comminuted. Furthermore, bilateral pterygoid plates fractures and bilateral comminutions of zygomatic buttresses and frontal walls of maxillary sinuses extending to inferior orbital rim and nasofrontal suture were observed. Fractures in left zygomatico‐sphenoid and zygomaticofrontal suture and left zygomatic arch were depicted. Also, CT scan imaged a Naso‐Orbito‐Ethmoidal (NOE) fracture. Moreover, a unilateral coronoid process fracture just above the mandibular notch was noted on the right side. Final diagnosis for the patient consisted of Lefort II, left zygomatico‐maxillary complex (ZMC), NOE type 3b, left orbital blow‐out, and right mandibular coronoid fractures [21] (Figure 2).

**FIGURE 3:**
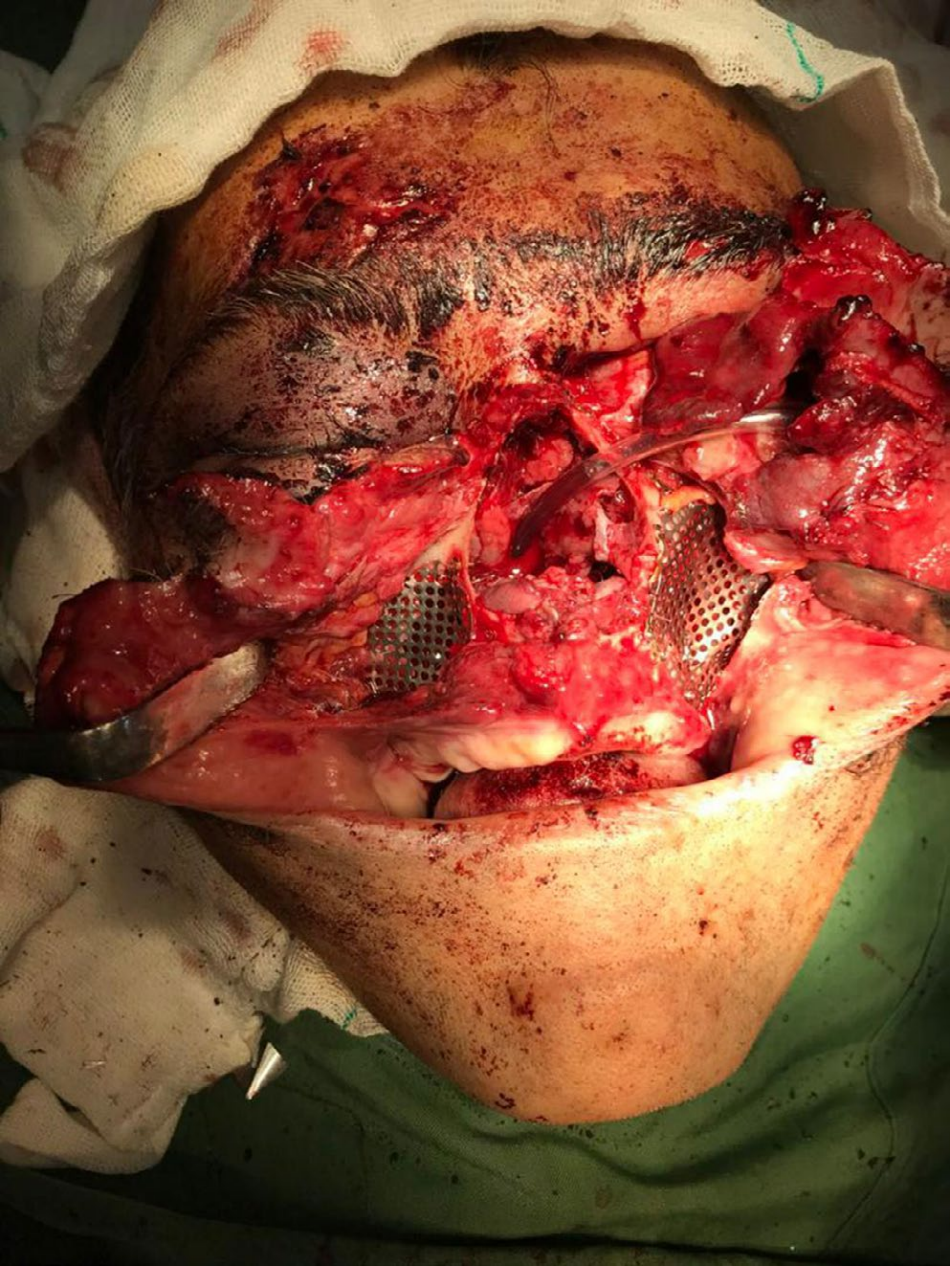
Two nelaton catheters in nostrils for maintaining airway patency

**FIGURE 4:**
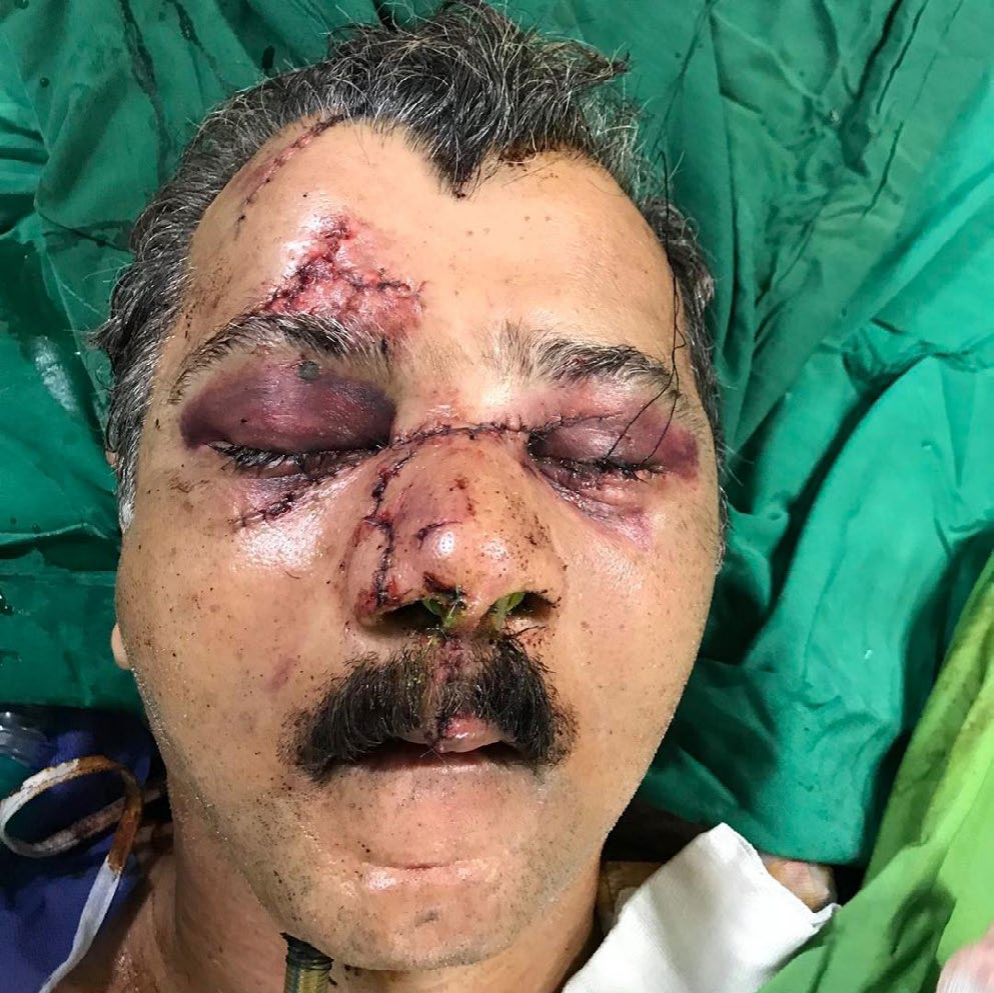
Immediate postoperative picture depicting closure of soft tissue lacerations

**FIGURE 5:**
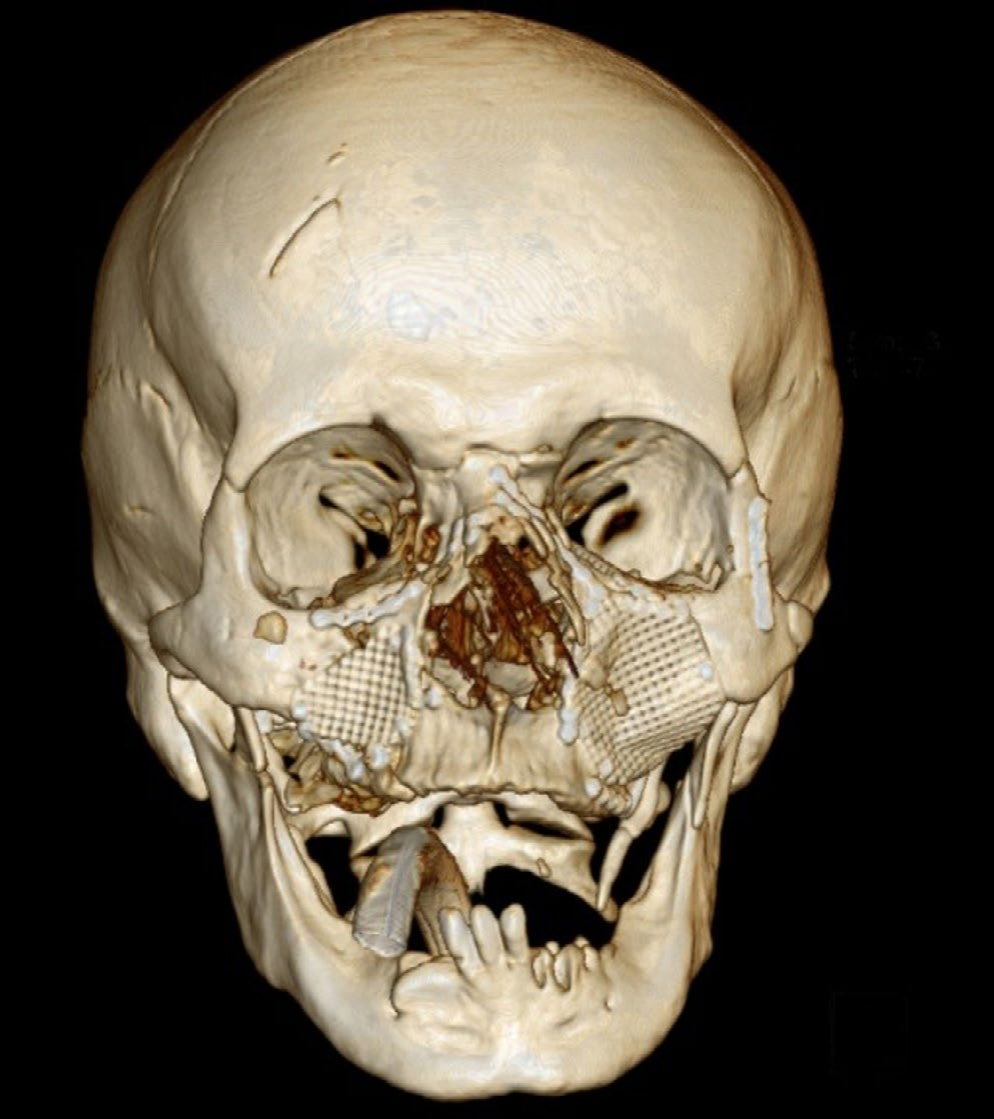
Immediate three‐dimensional CT scan depicting reduction of fractures using miniplates and screws and utilization of titanium mesh for reconstruction of orbital floor and anterior walls of maxillary sinuses

Patient went under general anesthesia through submental endotracheal intubation. Surgical access was obtained through expansion of existing lacerations for visualization of underlying skeletal structures. Following a thorough and rigorous irrigation with saline, airway patency was maintained using two nelaton catheters in nostrils (Figure 3). After a laborious 9‐hour surgery, comminuted structures including left orbital floor and bilateral frontal walls of the maxillary sinus were reconstructed by titanium mesh plates and the remaining fractured structures were reduced and fixed using microplate and screws in an outside‐to‐inside fashion and a primary stabilization of mid‐facial structures was obtained. Both eyes were salvaged. Suturing of the lacerations was accomplished except the nose with the nelaton catheters inside the nostrils. Nelaton catheters were removed and suturing of the nose was completed in final stage. Postoperative clinical image and 3D‐reconstructed CT scan are shown in Figures 4 and 5.

He was admitted for seven days in the hospital and then subsequently discharged. During postoperative period, no complications including infection, wound dehiscence, or retrobulbar hemorrhage were reported.

**FIGURE 6:**
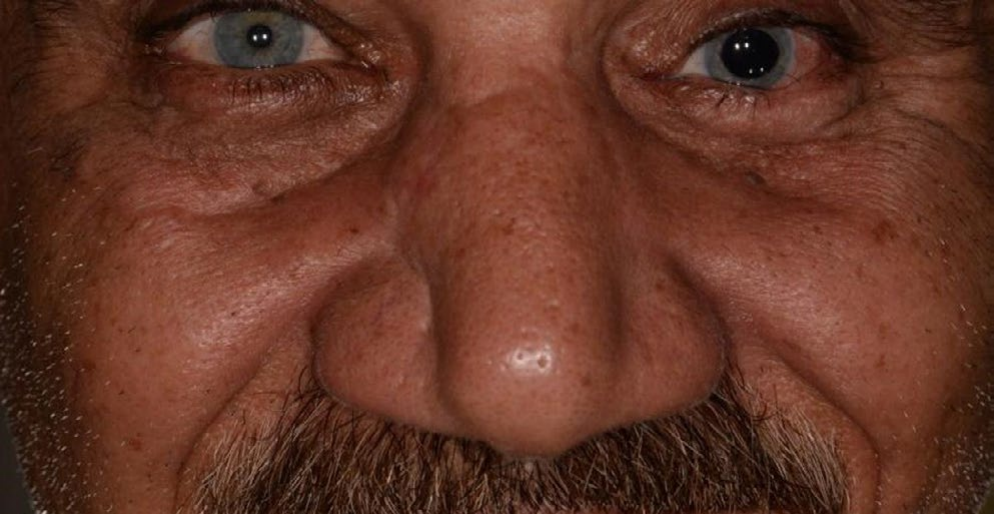
Anisocoria evident in patient’s right eye

Ocular examination after surgery revealed anisocoric. Normal findings in the right eye and decreased vision accuracy in the left eye were reported (Figure 6).

**FIGURE 7:**
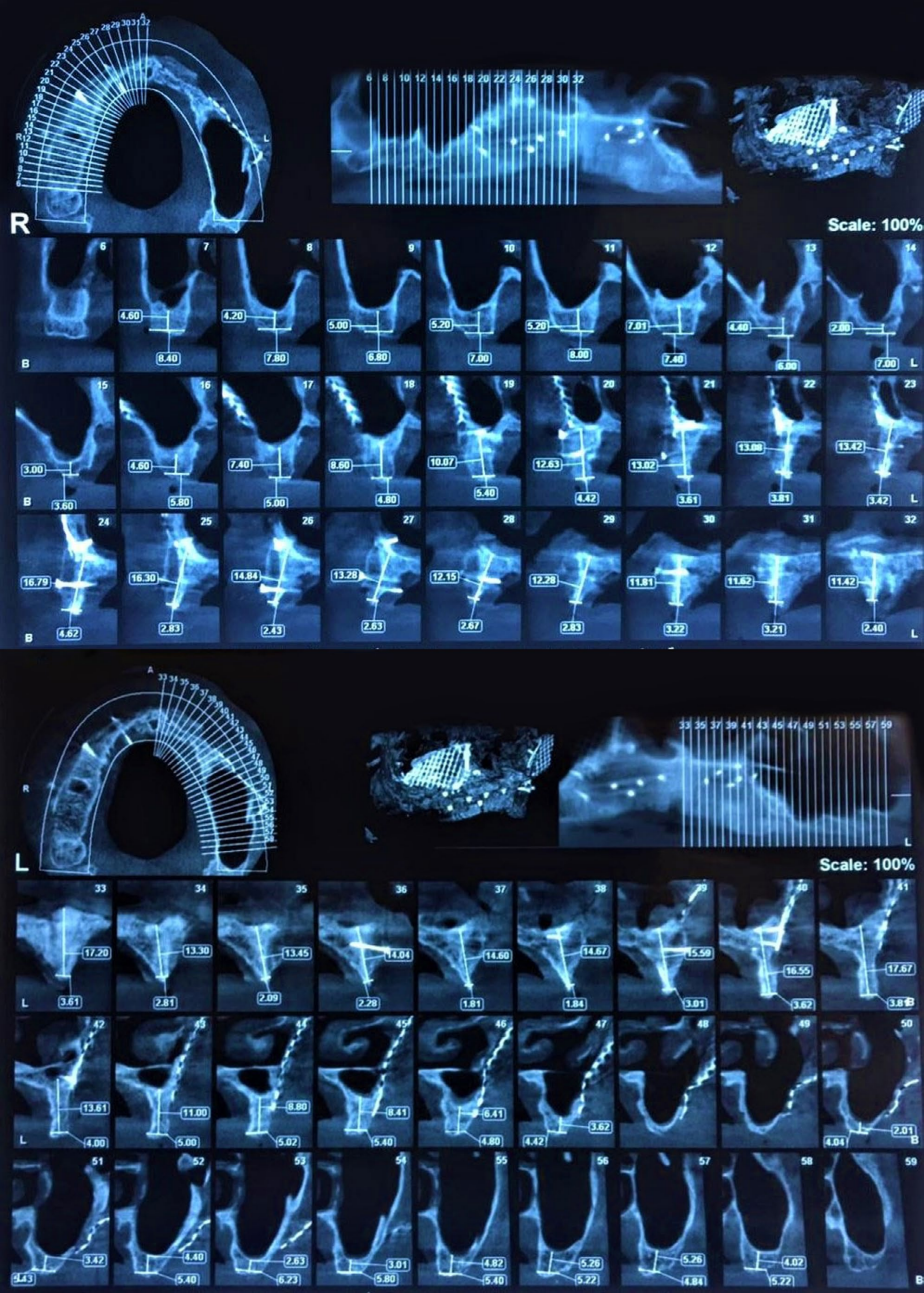
Preoperative cone beam computed tomography (CBCT) scan of the maxilla revealing inadequate remaining crestal bone necessitating crestal ridge augmentation for implant placement

**FIGURE 8:**
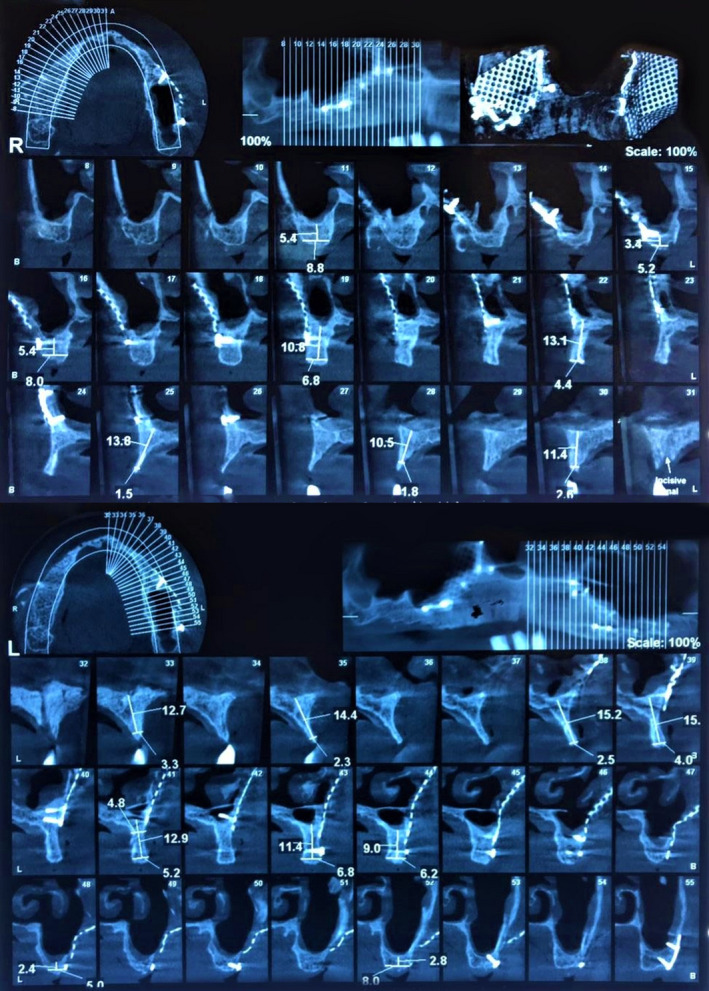
Cone beam computed tomography (CBCT) scan of augmented maxillary crestal ridge prior to implant placement

**FIGURE 9:**
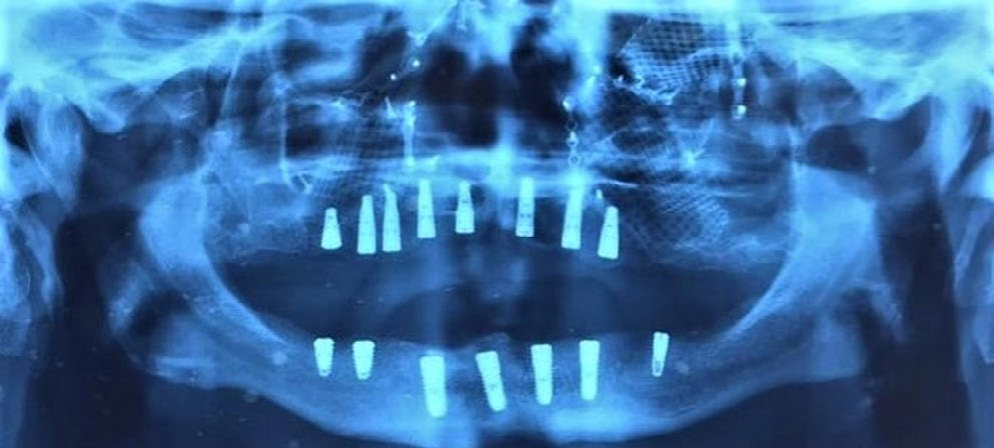
A total of 15 implants (7 in mandible, 8 in maxilla) were placed following crestal ridge augmentation of maxilla

**FIGURE 10:**
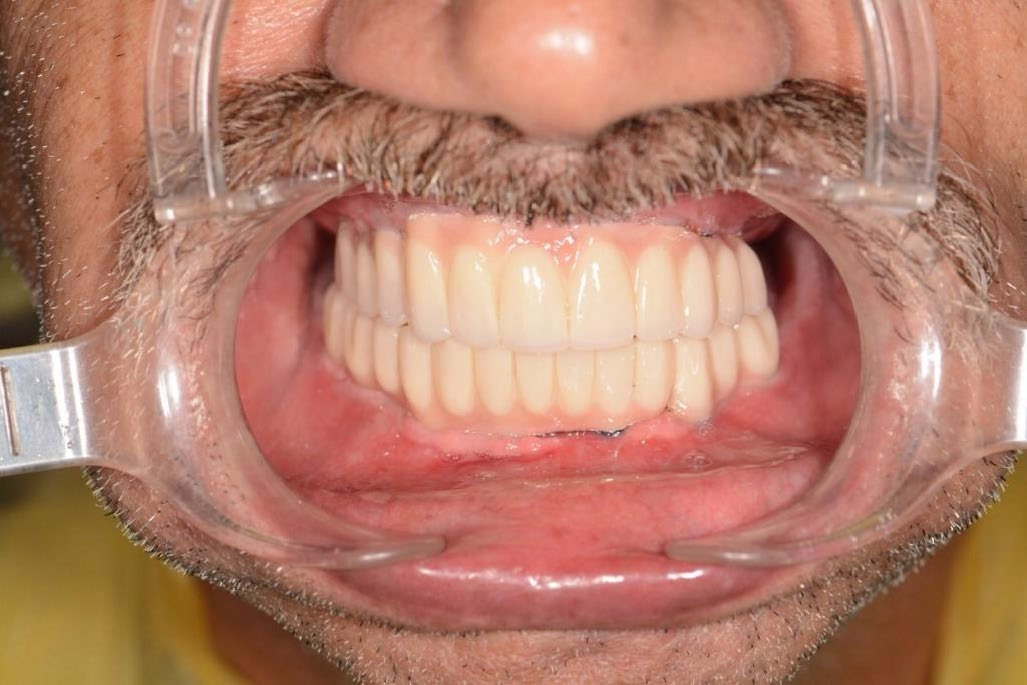
Mandibular and maxillary fixed prosthesis were delivered 4 months after implant placement

**FIGURE 11:**
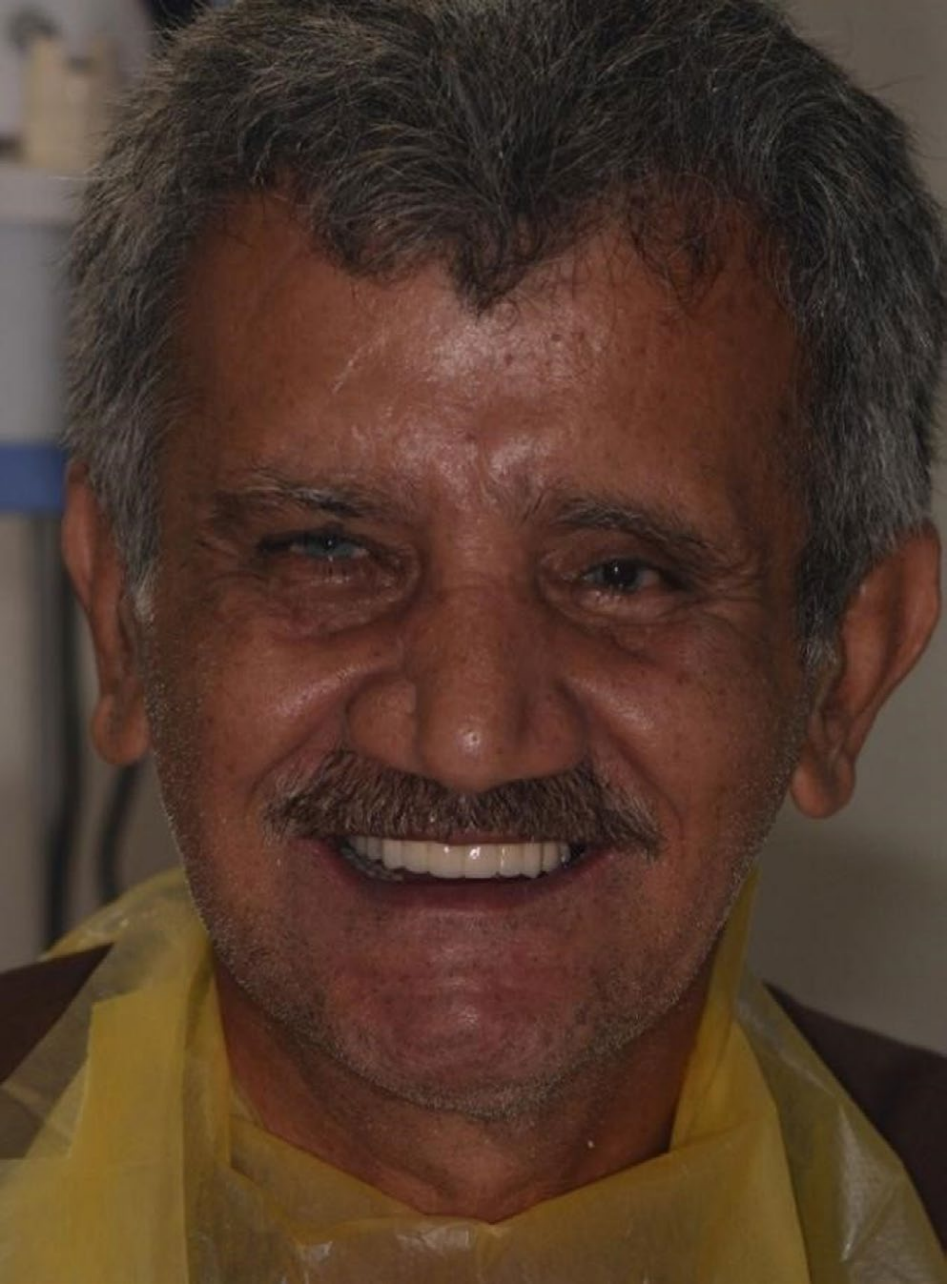
Final physiognomy of the patient after delivering his prosthesis

Following a year after the initial surgery, the patient referred to us seeking rehabilitation of the edentulous areas in his mouth. He used a maxillary complete denture and mandibular partial denture that were broken in the accident. Cone beam computed tomography (CBCT) and orthopantomogram (OPG) were then obtained which revealed inadequate bone width in the maxilla and therefore necessitating a crestal bone augmentation and bone grafts (Figure 7). Initial treatment plan consisted of fixed implant prosthesis on maxilla and removable overdentures in the mandible. Oral rehabilitation was scheduled for this patient in three stages. In the first stage and under general anesthesia, the reconstruction of maxillary atrophic ridge was done using autogenous bone graft harvested from anterior iliac spine. The remaining four mandibular teeth were then extracted and 4 implants (Osstem^®^ fixture, TS III SA) inserted immediately during this stage. During the healing period, no infection, wound dehiscence, or tenderness was observed on the graft recipient or donor sites. After 6 months, patient recalled for implant insertion with a new CBCT of augmented sites (Figure 8). Therefore, 8 maxillary implants (Neodent^®^ Acqua Drive) were inserted in augmented sites. After a few early follow‐up sessions, the patient decided to have a fixed prosthesis for the mandible as well, therefore, 3 additional implants (Osstem^®^ fixture, TS III SA) were placed in the mandible (Figure 9). After 4 months, maxillary and mandibular fixed prosthesis was delivered to the patient (Figures 10 and 11).

## 3 DISCUSSION

Maxillofacial traumas have been associated with 14%‐17% of all facial injuries ^6^,however, the incidence of severe or complex maxillofacial trauma seems to have decreased over the past 10 years ^7^. The most common etiology of mid‐facial traumas is motor vehicle accidents followed by interpersonal assaults ^8^. These traumas more commonly occur in males rather than females and most frequently in the second and third decades of the life as a result of motor vehicle accidents, assaults, falls, and domestic or occupational accidents ^9^. Global trends tend to show an increasing male/female ratio, specifically in societies where women are mostly confined to home ^10^.

The devastating nature of maxillofacial defects makes reconstruction of the maxilla and mandible challenging, due to multiple required surgeries and extensive rehabilitation phase. These patients often suffer signs and symptoms consistent with anxiety, depression, or post‐traumatic stress disorder ^11^. Restoration of the structural integrity as well as rehabilitation in order to retain functional and esthetic demands of the patient should be the primary goal of treatment ^12^, ^13^.

All patients presenting with severe facial traumas should be managed according to Advanced Trauma Life Support (ATLS) guidelines. Intracranial, cervical spine, thoracic, abdominal, and other severe extremity injuries must be ruled out or managed before tackling the facial reconstruction ^5^, ^14^, ^15^.

High‐definition computerized tomography (CT) scans with thin slices and three‐dimensional reconstruction are invaluable in examination, treatment planning, and long‐term management of facial traumas and have become a necessity in today’s management modalities of facial traumas ^16^.

The face is composed of three vertical and three horizontal buttresses which play an effective role in distributing and absorbing the forces of induced trauma in order to prevent them from affecting the brain. Properly aligned skeletal buttresses give structural and functional stability and integrity to the middle third of the face. Therefore, proper reconstruction of these key components of the midface is imperative ^5^.

In this case, we used submental intubation, as it is safe and easy to achieve without the need of any specialized equipment. Furthermore, it causes no interference in achieving occlusion intraoperatively and reducing the compartments of the midface. Surgical access was obtained through expansion of existing lacerations for visualization of underlying skeletal structures. In order to maintain nasal airway patency, two nelaton catheters were inserted in nostrils. Comminuted left orbital floor was totally reconstructed with titanium mesh. Also, bilateral frontal walls of the maxillary sinus were reconstructed by titanium mesh plates. The remaining fractured structures were reduced and fixed using microplate and screws in an outside‐to‐inside fashion and a primary stabilization of mid‐facial structures was obtained.

One of the main consequences of maxillofacial traumas is destruction of the teeth and teeth bearing alveolar bone. Oral rehabilitation utilizing dental implants of these patients must be carried out according to the following concepts: 1. the biological and anatomical features relative to the bone tissue to be treated with surgery; 2. utilization of a minimally invasive surgical techniques; 3. optimal management of peri‐implant soft tissues; 4. evaluation of the shape and surface geometry and the type of dental implant required; 5. ensuring proper placement and alignment of the implant in the bone crest ^17^. A key determining factor for a proper osseointegration of implants is to have a quantity of bone that measures at least 2 mm around the implant ^18^, ^19^. In this case, we scheduled a three‐stage oral rehabilitation plan including maxillary ridge augmentation with autogenous iliac bone graft and maxillary and mandibular implant‐supported fixed prosthesis.

## 4 CONCLUSION

In this study, we reported the management and further oral rehabilitation of a case suffering severe midface trauma following a motor vehicle accident where the patient was hit by a lorry. After reconstruction of midface fractures in the first stage, oral rehabilitation was successfully accomplished in three stages for the patient with satisfactory outcomes.

## CONFLICT OF INTEREST

The authors declare no conflict of interest.

## AUTHOR CONTRIBUTION

A.P. and M.A. conducted the surgery. K.A. was responsible for the manufacturing of the prosthesis. R.D., H.M., and S.S. were responsible for data collection and writing of the manuscript. R.D. was responsible for editing and revising of the manuscript. All authors discussed the results and contributed to the final manuscript.

## ETHICAL STATEMENT

Ethical approval by Ethics Committee of School of Dentistry, Shahid Beheshti University of Medical Sciences.

## INFORMED CONSENT

Written informed consent was obtained from the patient for the publication of his medical history, pictures, and outcomes in this report.

## Data Availability

The data that support the findings of this study are available from the corresponding author upon reasonable request.
